# Practical Notes on Diagnosis and Treatment

**Published:** 1906-06-09

**Authors:** 


### Practical Notes on Diagnosis and Treatment.

### Ménière's Disease.

"In Ménière's disease the methods of treatment to be considered are first Weir Mitchellism, secondly hydrobromic acid, and in the third place anti-gouty remedies.—*Sir Victor Horsley.*

### Angina Pectoris.

"I have never known a patient to lie flat in angina [pectoris] and it is very unusual for him to complain of palpitation."—*Sir Wm. Broadbent.*

### Vertigo.

According to Dr. Dundas Grant, recent experiments by Dreyfus show that quinine has a sedative action on the vestibular nerve. It is not necessary to give doses sufficient to cause deafness. Much smaller doses are quite sufficient to produce a favourable influence in the treatment of vertigo. In a recent lecture on "Aural Vertigo," Dr. Hughlings Jackson gave a similar recommendation.

### Corns and Warts.

Corns and warts can be successfully treated by the local application of sea-water or of a solution of "sea-salt." A bath of ten to fifteen minutes' duration twice daily for two to three weeks is usually sufficient. In the case of warts on the scalp a compress with sea-water should be applied and kept in position during the night.—*Dr. Arthur Evershed.*

### Blood Examination in Cancer.

Most cancerous patients are anæmic, and a blood count shows that the type is that of a so-called secondary anæmia. In some cases of cancer of the stomach the red corpuscular count is normal or higher than normal, this being usually due to persistent vomiting, or to a secretion of a large quantity of fluid into the stomach.—*Dr. John Fawcett.*

### Secondary Cancer.

Tumours of the bone secondary to cancer of the breast may develop merely with symptoms of pain and weakness, without there being any definite tumour to be felt.—*Mr. Anthony A. Bowlby.*

### Tuberculous and Non-Tuberculous Pleurisy.

If the fluid in tuberculous pleurisy is large in quantity and shows no sign of absorption it had better be removed. There does not appear to be any greater danger in removing the fluid in tuberculous cases than in non-tuberculous. The fluid in non-tuberculous pleurisy is either absorbed or, if removed by paracentesis, does not as a rule re-accumulate to any great exent. Two tappings are rarely if ever necessary in non-tuberculous pleural effusion. —*Dr. Sidney Martin.*

### Prevention of Abortion.

"To patients with threatened abortion you may give . . . chlorate of potassium . . . Sir James Simpson's directions for giving it are to administer 20-grain doses three times daily on an empty stomach, and it may be pleasantly taken in aerated water."—*Dr. F. J. McCann.*

### Sedatives in Pneumonia.

"There are cases of acute pneumonia in which continued pain, insomnia, or delirium is a serious danger . . . and it may be justifiable to give morphine, chloral, or other sedative. Personally I should prefer to give $\frac{1}{200}$th grain of hyoscine, and to repeat it in an hour or two if necessary. For violent maniacal delirium, the late Dr. George Balfour's prescription of half a dram of hydrate of chloral and 20 minims of tincture of digitalis sometimes succeeds admirably."—*Dr. C. H. Cattle.*

### Pilocarpine in Kidney Disease.

Pilocarpine is a most useful drug in restlessness, sleeplessness, or threatening uræmia in the third stage of granular kidney; it is not attended by the risks which are sometimes mentioned.—*Dr. Samuel West.*

### Croton-Oil Liniment in Sciatica, etc.

Croton-oil liniment will often succeed in cases of chronic sciatica and allied disorders in the hip region; thorough rubbing in has much to do with the success of the remedy. Used in this fashion, also, the same remedy seldom fails to relieve the stiffness and gnawing pains of rheumatic arthritic cases.—*Dr. J. Farquhar.*

### Corneal Opacities.

No method employed for the purpose of clearing up corneal opacities can show results comparable to those produced by dionine. The more recent the opacities the better the results, but even those of old standing show improvement. An ointment is the best preparation to employ. A little is placed on the eyeball, and the cornea is gently massaged through the closed lids, at first once, but afterwards twice daily. To commence with, the ointment may have a strength of 4 grains to the ounce, and this may be gradually increased up to 12 grains to the ounce. With active inflammation (*e.g.* in interstitial keratitis) dionine (1 to 2 per cent.) may be added to the atropine ointment which such a condition demands.—*Dr. Jas. Hinshelwood.*

### Chronic Colitis in Infants.

A mixture containing the yolk of one egg, an ounce of glycerine, one or two ounces of olive oil, and 10 to 15 minims of creasote, is most useful. One or two teaspoonfuls may be given three times daily for a long period. The creasote prevents the mixture going bad and may have some beneficial influence on the ailmentary canal.—*Dr. Edmund Cautley.*

### Calcium Chloride in Hæmorrhage.

Calcium chloride is now generally recognised as an agent which promotes the coagulating power of the blood and is thus used to hceck, and to prevent hæmorrhage. But, acording to Professor A. E. Wright, if given in excess it will produce exactly the opposite effect. It should never be prescribed for more than three or four days in continuous administration.

